# Pathosphere.org: pathogen detection and characterization through a web-based, open source informatics platform

**DOI:** 10.1186/s12859-015-0840-5

**Published:** 2015-12-29

**Authors:** Andy Kilianski, Patrick Carcel, Shijie Yao, Pierce Roth, Josh Schulte, Greg B. Donarum, Ed T. Fochler, Jessica M. Hill, Alvin T. Liem, Michael R. Wiley, Jason T. Ladner, Bradley P. Pfeffer, Oliver Elliot, Alexandra Petrosov, Dereje D. Jima, Tyghe G. Vallard, Melanie C. Melendrez, Evan Skowronski, Phenix-Lan Quan, W. Ian Lipkin, Henry S. Gibbons, David L. Hirschberg, Gustavo F. Palacios, C. Nicole Rosenzweig

**Affiliations:** Biosciences Division, Edgewood Chemical and Biological Center, 5183 Blackhawk Rd, Aberdeen Proving Ground, Edgewood, MD 21010 USA; OptiMetrics, Inc, Abingdon, MD USA; Center for Genome Sciences, United States Medical Research Institute of Infectious Diseases, Ft. Detrick, Frederick, MD USA; Department of Biomedical Informatics, Columbia University, New York, NY USA; Walter Reed Army Institute of Research, Viral Diseases Branch, Silver Spring, MD USA; TMG Biosciences LLC, Austin, TX USA; The Center for Infection and Immunity, Columbia University, New York, NY USA; Joint Genome Institute, Department of Energy, LBNL, Berkley, CA USA; Department of Interdisciplinary Arts and Sciences, University of Washington Tacoma, Tacoma, WA USA

## Abstract

**Background:**

The detection of pathogens in complex sample backgrounds has been revolutionized by wide access to next-generation sequencing (NGS) platforms. However, analytical methods to support NGS platforms are not as uniformly available. Pathosphere (found at Pathosphere.org) is a cloud - based open - sourced community tool that allows for communication, collaboration and sharing of NGS analytical tools and data amongst scientists working in academia, industry and government. The architecture allows for users to upload data and run available bioinformatics pipelines without the need for onsite processing hardware or technical support.

**Results:**

The pathogen detection capabilities hosted on Pathosphere were tested by analyzing pathogen-containing samples sequenced by NGS with both spiked human samples as well as human and zoonotic host backgrounds. Pathosphere analytical pipelines developed by Edgewood Chemical Biological Center (ECBC) identified spiked pathogens within a common sample analyzed by 454, Ion Torrent, and Illumina sequencing platforms. ECBC pipelines also correctly identified pathogens in human samples containing arenavirus in addition to animal samples containing flavivirus and coronavirus. These analytical methods were limited in the detection of sequences with limited homology to previous annotations within NCBI databases, such as parvovirus. Utilizing the pipeline-hosting adaptability of Pathosphere, the analytical suite was supplemented by analytical pipelines designed by the United States Army Medical Research Insititute of Infectious Diseases and Walter Reed Army Institute of Research (USAMRIID-WRAIR). These pipelines were implemented and detected parvovirus sequence in the sample that the ECBC iterative analysis previously failed to identify.

**Conclusions:**

By accurately detecting pathogens in a variety of samples, this work demonstrates the utility of Pathosphere and provides a platform for utilizing, modifying and creating pipelines for a variety of NGS technologies developed to detect pathogens in complex sample backgrounds. These results serve as an exhibition for the existing pipelines and web-based interface of Pathosphere as well as the plug-in adaptability that allows for integration of newer NGS analytical software as it becomes available.

**Electronic supplementary material:**

The online version of this article (doi:10.1186/s12859-015-0840-5) contains supplementary material, which is available to authorized users.

## Background

**Fig. 1:**
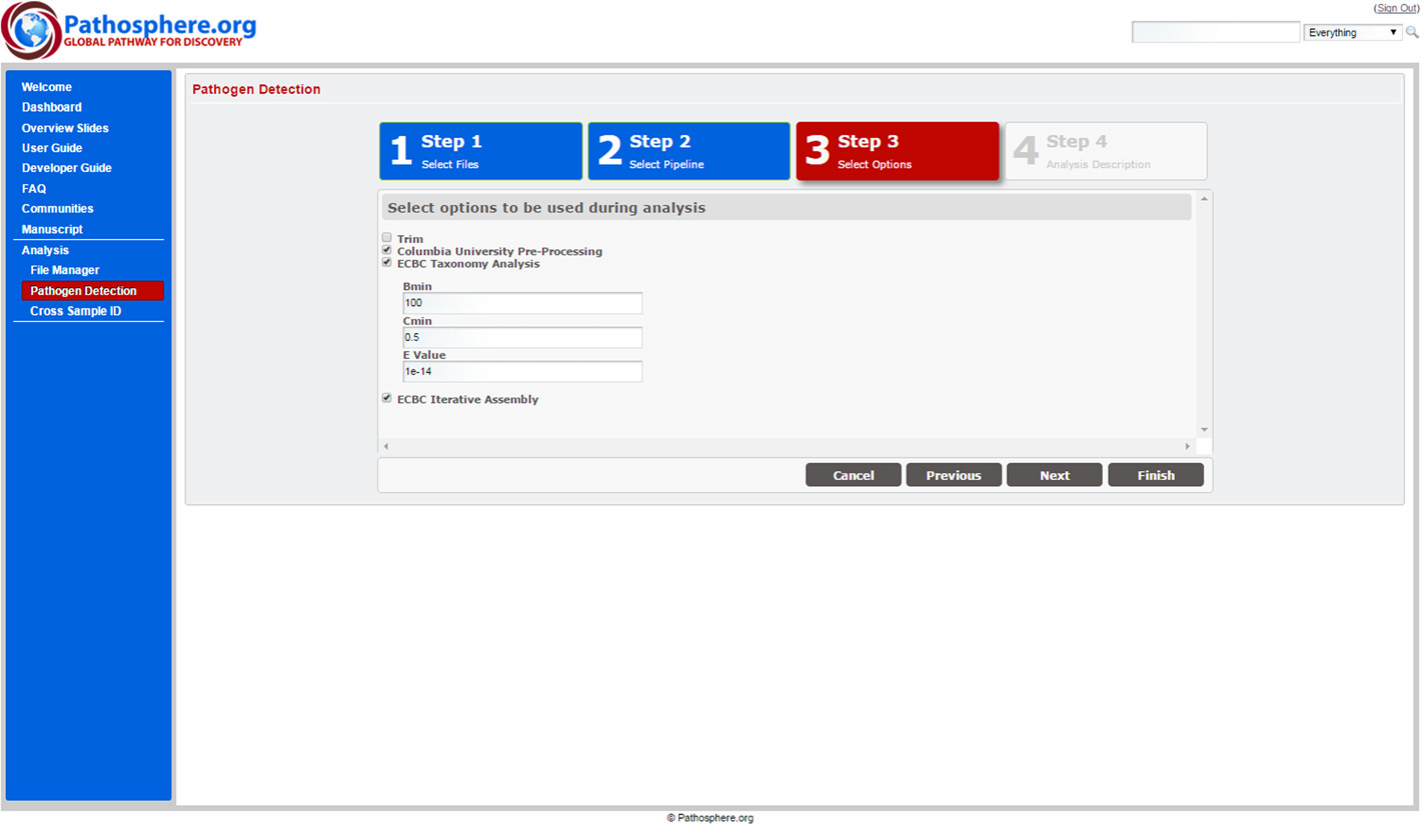
Pathosphere user interface. The web-based portion of Pathosphere contains message boards, forums, user communities to share data and results, a live-chat messager, user and developer guides and FAQs, as well a custom interfaces for the pathogen detection pipelines utilized by the current Pathosphere users. This screenshot displays the user-defined parameters that are customizable for each pathogen detection run

The increasing availability of next-generation sequencing (NGS) platforms has allowed for NGS technology to play a critical role in molecular biosurveillance and outbreak management [1–4]. NGS techniques can give an unparalleled depth and range of detection in samples containing unknown pathogens. However, using NGS platforms for these applications requires not only sequencers and personnel to generate high quality and reliable sequencing data, but also the means to organize and interpret the large data sets generated. Analysis typically requires significant investment in computer hardware, analytical software, and technical support. The website Pathosphere (pathosphere.org) was created to provide both the hardware and software capabilities necessary to detect pathogens in NGS data (Fig. 1). By creating a web-based capability, analysis and computational resources can be shared widely with direct engagement of the crowd-sourced biosurveillance community.

Next - generation sequencing technology has the potential to give an unbiased, in-depth snapshot of what exists in a sample. Currently, the analysis of the data generated from NGS platforms can be a limiting factor for pathogen detection. Identifying the pathogen sequences represented within large data sets is difficult both from the perspective of the hardware and software requirements. The rise of open source software and cloud computing has supported the proliferation of capabilities developed for NGS data analysis. Recently developed computational analyses [2, 5–8] can detect pathogens from samples derived *in silico* as well as from samples with potentially complex backgrounds. However, there is a need for local hardware support to run these analyses or the cloud-based availability for the hosting of software.

Pathosphere is a free service designed to provide the larger bioinformatics community a means to source their software. Current analytical capabilities include background taxonomic analysis of read files, sequence assembly, pathogen identification using databases such as NCBI, and reports that are easy to interpret. To facilitate pathogen detection by laboratories or entities that do not possess the hardware components or technical staff necessary for the process-heavy data analysis from NGS sequencing platforms, the Pathosphere interface allows researchers to perform sequence data analysis globally by uploading data to a hosted cloud portal [9]. Pathosphere also supports analytical automation, which allows for non-heavy users to upload data and then receive generated reports upon the completion of a chosen pathogen identification pipeline. While these pipelines are automated, the values used for pre-processing and analysis can be adjusted from the recommended defaults, adding another layer of flexibility for certain targeted applications that might be desired for genomic data analysis. Analytical tools can be added by the community, and the plug-in compatibility of the Pathosphere architecture allows for the addition of new open-source software to be integrated seamlessly over time. This design will ensure that Pathosphere evolves as newer and improved analytical software and methods are developed. Pathosphere is designed to allow for collaboration within groups, but also securely stores the communications and data that are uploaded for sharing and analysis. To date, Pathosphere has 192 separate user accounts, with 79 users posting 1,450 comments in 31 communities. Pathosphere has been visited over 5,000 times since its inception.

In this study, Roche 454 pyrosequencing, Ion Torrent, and Illumina data were generated from varying sample types as a test of Pathosphere architecture and function. The capabilities of the Pathosphere pipelines to analyze 454, Ion Torrent, and Illumina data generated from an identical sample were compared, and then the Pathosphere analytical pipelines were tested for their ability to identify pathogens in diverse sample types in which no pathogen was detected using traditional methods. Finally, the flexibility of Pathosphere was demonstrated by integrating another analysis pipeline to do follow-on analysis of pathogenic samples not recognized initially. The evaluation of the pathogen identification and analysis pipelines provided by Pathosphere will serve to introduce the capabilities of Pathosphere while also highlighting gaps which the emerging infectious disease community can address in the future.

## Methods

### Pathogen isolate sample preparation

#### Isolates sample preparation

Samples 712 and 808 containing LuJo virus were prepared from human isolates [10]. RNA was extracted from the cerebrospinal fluid and serum of a liver transplant recipient. After digestion with DNase I to eliminate human chromosomal DNA, RNA preparations were amplified by means of reverse-transcriptase PCR (RT-PCR) with the use of random primers [11, 12]. Amplification products were pooled and sequenced with the use of the 454 Genome Sequencer FLX platform (Roche, Branford, CT), but DNA fragmentation was omitted. The Zaria bat coronavirus samples 819 and 820 (and the negative control 806) were obtained from the GI tract of bats that tested positive (and negative for the control) for coronavirus by PCR [13]. Sample 28 containing GBV-D was obtained from bat serum [14] and prepared as detailed previously. The isolated RNA for both coronavirus and GBV-D samples was converted to cDNA and the library was prepared similarly to the LuJo virus isolates detailed above. The bat parvovirus sample, 1164, was obtained from the spleen of parvovirus PCR-positive bats (like those discovered in [15, 16]), and DNA was isolated and the prepared libraries were sequenced on the 454 FLX (Roche, Branford, CT). Samples containing MERS-CoV (1500, 1501) [17] were prepared as previously described [18]. Viral cDNA was made using random primer RT-PCR from nasal swabs of camels. Further PCR amplifications were made using overlapping PCR primers spanning 2.0–2.5 kb fragments of MERS-CoV [19]. These amplification products were pooled and sequenced on the Ion Torrent PGM platform. The human serum spiked samples containing *Y. pestis, F. tularensis, and B. anthracis, ​B. mallei, and B. psuedomallei* were prepared for sequencing as described previously [20, 21] and sequenced on 454 FLX (Roche, Branford, CT), Ion Torrent PGM (Life Technologies, Grand Island, NY), and Illumina MiSeq platforms (Illumina, San Diego, CA). SRA information for each sample analyzed here are available through the NBCI BioProject # PRJNA276557.

## Implementation

### ECBC pipeline

**Fig. 2:**
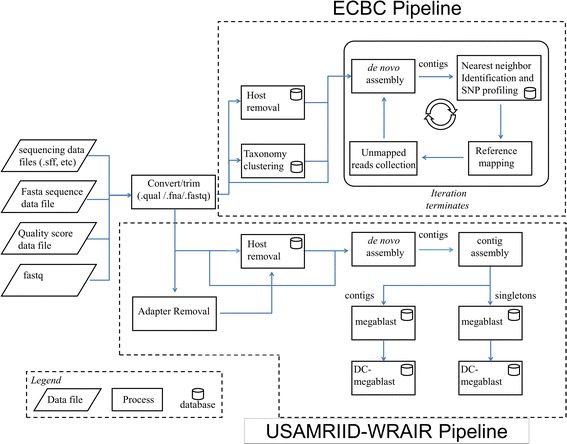
Summary of the analytical capability of the bioinformatics pipeline. Data can currently be preprocessed by two tools, Columbia University’s Preprocessing Procedure (CUPP) or a taxonomy analysis based on NCBI taxonomy results. Then, reads retained after the pre-processing manipulations are assembled *de novo*. Nearest neighbors and SNP profiling then occurs by comparing the identified contigs to NCBI databases. A reference map is created, and the SNP profile from those mapping results provides a comprehensive comparison of the taxonomical near neighbors. Finally, all the unmapped reads are extracted and used as input to the next iteration

The pipeline described below was designed to integrate a wide range of analytical tools into a single automated process (Fig. 2)

.

NGS data is first run through quality control trimming using standard metrics as the default but allowing for user trimming flexibility. Two preprocessing tools are currently available; Columbia University’s Preprocessing Procedure (CUPP) and a taxonomic analysis based on NCBI taxonomy results. CUPP was developed to reduce the complexity and total size of a NGS dataset. In this procedure, all the reads in the sample are compared using bowtie2 [22] to map reads against the CUPP database and then remove host reads from the analysis. The host databases for CUPP include *Anopheles gambiae* (mosquito), *Danio rerio* (zebra fish), *Gallus gallus* (chicken), *Homo sapiens* rRNA (human), *Homo sapiens* chromosome (human), *Mus*_musculus (rodent), *Sus scrofa* (pig), mitochondrion genome, and *Xenopus laevis* (frog) . The taxonomy analysis provides a lowest common ancestor for each read, thus providing a general description of bacterial, viral, and eukaryotic constituents in the sample. These procedures, CUPP and taxonomy analysis, can be used individually or serially as part of an analysis request (Fig. 1). These tools, and the code used to implement them into the analytical pipeline, are available as open-source software at (http://sourceforge.net/projects/pathosphere/?source=directory).

The iterative analysis is designed to identify pathogens without assumptions about the sample identity or complexity. To fulfill this goal, a process has been constructed to perform a subtractive approach in searching for possible multiple pathogens or multiple chromosomal elements in a single sample. First, the genomic data uploaded to the system, or reads retained after the pre-processing manipulations, are processed through a *de novo* assembly. In the case of 454 data, the reads are assembled using the GS Newbler (Roche) program [23]. For Illumina data, the reads are assembled with velvet [24]. The *de novo* assembly produces longer contiguous lengths (contigs) of genomic sequences. A database search step then compares the contigs with genome sequences in the NCBI nt database to identify high quality matches. Each query (from a *de novo* assembled contig) results in a series of hits which are ranked by BLAST bit score. The resultant top hit per query is cumulatively ranked using bit score compared to the other top hits. The topmost ranked NCBI database genome sequence in the cumulative ranked list is selected as the nearest neighbor (NN) sequence for the iteration.

In the next step, the taxonomical neighbors of this NN in the NCBI nucleotide database are collected according to the following procedure: if NN is ranked as subspecies, or its direct taxonomical parent is ranked as subspecies, all the database records belonging to the same NCBI taxonomic subspecies sub-tree are collected; if the total count of the collected records is less than 20 (default value, can be reset by user), then the species sub-tree the NN belongs to is searched and the additional database records that belong to this sub-tree (and that also appear in the rank hit list) are collected. After the NN's neighbor genomes are collected, all the input reads for this iteration are mapped to each of those genome sequences by reference mapping.

In the final step of the first iteration, all the input reads used for *de novo* assembly are reference mapped to the NN reference, and the unmapped reads are extracted and used as input to the next iteration. For 454 data, the reads are referenced mapped using the GS Newbler (Roche) program [23]. For Illumina data, the reads are reference mapped with the Bowtie2 program [25]. In the next iteration, the steps described above are repeated. The iterative analysis allows multiple chromosomes, plasmids, or inserted genomic elements to be identified and reported to the user for directed, manual analysis.

### USAMRIID-WRAIR pipeline

The USAMRIID-WRAIR pipeline was designed to be modular and thus give it flexibility to integrate new software as it becomes available, replacing older versions for reasons such as speed and sensitivity. Acceptable input formats include SFF, fastq single or paired-end, and compressed gzipped files. Step1 first decompresses the file and/or converts the file into fastq format if an SFF file is the starting input. The converted fastq or paired-end fastqs are processed for host removal using Bowtie2 [25]. The first iteration uses the host genome of choice for read removal followed by the host transcriptome. Once host reads are removed, adaptors are trimmed and reads go through quality filtering using cutadapt [26] and prinseq-lite [27]. Reads are assembled into contigs using the *de novo* assembler Ray Meta [28], followed by a contig assembly using Cap3 [29] to ensure the longest possible contigs.

Identification of contigs and single reads (singletons) is achieved through an iterative BLAST search using the NCBI nt database. Iterative BLAST 1 uses the contigs as the query and starts with a megablast followed by a discontiguous megablast. Only the contigs that do not get identified in the megablast go on to the dc-megablast. Iterative BLAST 2 is essentially the same except that the singletons are used as the input. These BLAST searching schemes ensure that highly homologous sequences (megablast: word size of 28) are matched appropriately, and that less homologous sequences (discontiguous megablast: word size 12) are identified within the dataset. The outputs are divided into contig and read reports. The output reports resemble a top blast output with the addition of reads that aligned to each contig. Taxonomy is assigned using names and nodes files from NCBI.

### Architecture and web implementation

Pathosphere is a practical implementation and reference design for scalable, secure web services for genomics processing. There are two main parts of the Pathosphere system. The first is a cloud-based web interface provided by custom applets running inside of Liferay (http://www.liferay.com/). The second part of the system consists of any number of backend processing computers or clusters. This architecture separates the web interface, user collaboration tools, and result display mechanisms from the systems that actually process the data through pipelines. In this way, the pipeline design, construction, execution, along with any hardware configuration, is completely independent from the server providing the user interface. This allows for unlimited flexibility in the types of pipelines being integrated into the Pathosphere system.

The cloud-based front end web server has relatively low system requirements, since this portion of the system only stores data and results, allows submission of jobs, and provides collaboration tools. This design keeps the computationally intensive processing tasks off of this server. Currently, as jobs are submitted, they are processed serially, although a more sophisticated job management system could be implemented. The current Pathosphere front end server resides on a single, mid-level server, but this portion of the system could be easily scaled up on more powerful servers if the user load were to increase in the future.

Like the front end web server, the backend servers in the Pathosphere architecture can also exist anywhere in the world with a network connection. These backend servers can range from single machines to large computational clusters, depending on the types of algorithms being processed. The pipelines described in this paper are set up to run on a computing cluster consisting of 14 blade servers, several supporting servers, and over 40 TB of shared storage. Similar to the front end, the backend processing needs are built to be expandable to cloud based services [9] when user load increases.

### Security features

Communication between the client and web server is via https, using TLS v1.0 or higher. The Public Key Infrastructure (PKI) certificate is a StartCom signed RSA 4096 bit key. This ensures secure communication between the client and the webserver. Individual users are authenticated using usernames and passwords. The only information stored about a run is its sample name and title. The user should not enter identifiable patient information in these fields, as the system is not intented to store confidential patient data. Only the data uploaded by a specific user is visible to that user, unless it is explicitly shared with another user. In order to join a community, a user must have permission from the group owner. The web server, mail server, and cluster all have network access restricted by external firewalls that limit access to only the expected network communication. The only access to the backend computing cluster is via a Secure Shell (SSH) connection, with a PKI key, ensuring that the data remains secure in transit. Data is not encrypted while stored on the computing cluster, but the cluster is located in a secure location on a military installation.

## Results

### Direct comparison of pathogen detection in 454, Ion Torrent, and Illumina sequenced samples using ECBC pipelines

To evaluate the pathogen identification capabilities of the ECBC pipeline with multiple types of sequencing data, a side-by-side comparison of three sequencing platforms was performed. A spiked human serum sample with *Y. pestis, B. anthracis, F. tularensis, B. mallei, and B. psuedomallei* was sequenced and subsequently analyzed using the ECBC pipeline on Pathosphere. When sequencing for pathogen identification, large amounts of background genomic material can complicate the analysis. To mitigate this, two preprocessing methods are available and used regularly as part of the analytical pipeline used for samples within complex backgrounds. CUPP systematically removes host background reads from common organisms. Human backgrounds are represented in this procedure, so CUPP is used as a preprocessing method for all samples evaluated in this section. Taxonomic analysis provides an indication of pathogens and near neighbors represented at lower concentrations: too low to produce an assembly. The source code for both preprocessing tools is open-source available on SourceForge (http://sourceforge.net/projects/pathosphere/?source=directory).

To directly compare the performance of the ECBC pipeline on three different sequencing platforms, a complex sample containing human serum spiked with *Y. pestis* (1x10^4^ CFU)*, B. anthracis* (1x10^6^ CFU)*, F. tularensis* (1x10^5^ CFU), *B. mallei* (1x10^2^), and *B. pseudomallei* (1x10^3^) was processed, sequenced on Roche 454, Ion Torrent, and Illumina MiSeq platforms, and then analyzed using the iterative analysis pipeline (Fig. 2). These data sets are also available on the Pathosphere homepage. The 454 and Ion Torrent files were similar in size, with the 454 raw data at 2.5GB and the Ion Torrent data at 1.6GB (Table 1). The Illumina data set was larger with 5GB in uncompressed paired-end read files. Both the 454 and Ion Torrent datasets ran through the complete pipeline at about the same rate, with the 454 data slightly slower at 35 minutes compared to the 28 min of the Ion Torrent data. The large size of the Illumina data set correlated with a longer analysis time, of 4 h and 11mins.

**Table 1:** ECBC Pipeline Analysis on Non-host Reads of Samples Containing *B. anthracis, F. tularensis, Y. pestis, B. pseudomallei, and B. mallei*. Unknown samples were created, sequenced on 454, Ion Torrent, and Illumina platforms and processed (methods). Datasets were then analyzed using the ECBC pathogen detection pipeline. Table shading represents the positive and correct identification of the organism listed. Unshaded cells represent the lack of single-read identification matching to the pathogens spiked

		454		Ion Torrent		Illumina	
		File size: 2.5gb		File size:1.62gb		File size: 5gb paired-read files	
		Pipeline runtime: 35m12s		Pipeline runtime: 29m18s		Pipeline runtime: 4hr11m	
Agent	Spiked Amounts	Taxonomy Assignment ID	Iterative Assembly ID	Taxonomy Assignment ID	Iterative Assembly ID	Taxonomy Assignment ID	Iterative Assembly ID
*B. anthracis*	1x10^6^ CFU						
*F. tularensis*	1x10^5^ CFU						
*Y. pestis*	1x10^4^ CFU						
*B. pseudomallei*	1x10^3^ CFU						
*B. mallei*	1x10^2^ CFU						

The taxonomy assignments and iterative assemblies identified pathogens within the samples sequenced by all three platforms with similar efficiency (Table 1). Taxonomy assignments identified *B. anthracis, F. tularensis, Y. pestis, and B. pseudomallei* in the 454 dataset, only *B. anthracis, F. tularensis, Y. pestis* in the Ion Torrent dataset, while all five pathogens were detected in the Illumina dataset (Table 1). Genomic elements (plasmids) of *Y. pestis* were detected in the early iterations of the 454 data while the genome sequence of *B. anthracis* and *F. tularensis* were detected in the later iterations (Additional file 1). A similar trend was observed with the Ion Torrent data, as the *Y. pestis* plasmid A1122 was detected in the first iteration, followed by the genomic sequences of *Y. pestis, F. tularensis, and B. anthracis*, respectively (Additional file 1). The Illumina dataset resulted in the detection of *Y. pestis* genome and plasmids for the first six iterations, followed by a genome *F. tularencis* assembly and two (genome and plasmid) *B. anthracis* assemblies (Additional file 1). Many of the reads sequenced by 454, Ion Torrent, and Illumina were able to be assembled into large contigs that mapped accurately and provided sufficient coverage to identify the pathogens within the sample (Table 1).

### Analytical pipeline identification of unknowns in complex samples using ECBC pipelines

The ECBC analytical pipelines on Pathosphere identified pathogens (chromosome and plasmids) within a spiked sample background, so Pathosphere was next evaluated using real-world samples containing pathogens. Variability in sample quality and pathogen levels can be complicating factors when attempting to detect pathogens within complex backgrounds using traditional methods as well as NGS technology [30–32]. In the following examples, human or animal material (serum, tissue, stool) containing pathogens difficult to detect using standard molecular techniques was prepared and sequenced (Table 2). The presence of pathogens in each sample used here was confirmed in other studies (see Methods) to validate correct pathogen identification during analysis on Pathosphere. These raw datasets are freely available to all Pathosphere members within a special tab located on the Pathosphere homepage (Fig. 1), along with all the detailed documentation.

**Table 2:** Viral Samples And Non-host Reads. Samples collected from various sources were sequenced for pathogen detection. CUPP removed host reads, leaving non-host reads for further iterative and taxonomic analysis. Samples obtained had already been confirmed to contain or not contain indicated virus (methods)

Sample	Host	Tissue	Viral Agent	Total Reads	non-host reads	non-host reads (%)
28	Bat (*Microchiroptera*)	Serum	Hepatitis G virus	69558	1704	2 %
712	*(Homo sapiens)*	Liver biopsy	Lujo virus	55227	759	1 %
806	Bat (*Microchiroptera*)	Gastro-intestinal tract	-	9308	4752	51 %
808	Human *(Homo sapiens)*	Liver biopsy	Lujo virus	43090	4479	10 %
819	Bat (*Microchiroptera*)	Gastro-intestinal tract	Zaria-CoV	29182	12450	43 %
820	Bat (*Microchiroptera*)	Gastro-intestinal tract	Zaria-CoV	67808	14790	22 %
1164	Bat (*Microchiroptera*)	Spleen	parvovirus	72877	34935	48 %
1500	Dromedary Camel (*Camelus dromedaries*)	Nasal Swab	MERS-CoV	924389	888708	96 %
1501	Dromedary Camel (*Camelus dromedaries*)	Nasal Swab	MERS-CoV	866598	831820	96 %

This pipeline detected the correct viral pathogens in all but one of the data sets, as shown in Table 3. Samples will be evaluated in this section in the following order: (1) True Negative (2) Pathogens detected through iterative assembly (3) Pathogens detected through taxonomic analysis and (4) Samples that demonstrate gaps in ECBC analytical pipelines on Pathosphere.

#### True negative

Sample 806 was selected as the negative control. Following CUPP, the *de novo* assembler failed to construct contigs from the pre-processed reads. To ensure that pathogens were not missed because assembly was unsuccessful, a taxonomy analysis was selected. Following the taxonomic analysis, assembly was still unsuccessful, and the taxonomic analysis revealed that none of the reads were positively identified as viral or bacterial in nature based on NCBI taxonomy. The taxonomy and prevalence of reads from different organisms help differentiate between sequencing artifacts, such as cross-contamination, and low-level infection.

#### Pathogens detected through iterative assembly

The iterative analysis reported the presence or absence of pathogen correctly in tissue and stool samples from both human and zoonotic hosts. Two samples in this category were previously reported as the old world arenavirus, Lujo virus [10, 33]. RNA was extracted from the liver biopsy sample, and amplification was pursued using random primers (Sample 712) and an additional rRNA depleting step (Sample 808). Both samples were analyzed with CUPP followed by iterative analysis to identify the pathogen. Two iterations were completed, identifying the two segments of the LuJo virus (Table 3). On the third iteration, the assembly failed to produce contigs thereby ending the analysis.

**Table 3:** Iterative Analysis on Non-host Reads of Sample 712, 806, 808, 819, 820, 1500, and 1501. Collected known samples (Table 2) were analyzed using the ECBC iterative analysis pipeline for pathogen detection. *De novo* assembled contigs are used to generate nearest neighbors, then the nearest neighbors are used to map reads and generate consensus contigs from the mapped reads (Fig. 2). Upon completion, a new iteration begins using reads not mapped to the nearest neighbor. The cycle completes after no further reads exist for contig building or there are no matches reported

Sample	Iteration	Reads	*de novo* Contigs	Nearest Neighbor Reported	Contigs Generated from Nearest Neighbor Read Mapping
712	1	759	3	Lujo virus segment S glycoprotein precursor and nucleocapsid protein genes, complete cds	2
	2	90	1	Lujo virus segment L multifunctional matrix-like protein and large RNA-dependent RNA polymerase genes, complete cds	1
806	1	4752	0	-	-
808	1	4479	4	Lujo virus segment L multifunctional matrix-like protein and large RNA-dependent RNA polymerase genes, complete cds	3
	2	1527	1	Lujo virus segment S glycoprotein precursor and nucleocapsid protein genes, complete cds	1
	3	139	0	-	-
819	1	12450	12	Zaria bat coronavirus strain ZBCoV, partial genome	1
	2	8247	9	-	no db hits
820	1	14790	31	Rhinolophus ferrumequinum clone VMRC7-267P18, complete sequence	1
	2	10222	37	Zaria bat coronavirus strain ZBCoV, partial genome	1
	3	9704	33	-	no db hits
1500	1	884863	3993	Middle East respiratory syndrome coronavirus complete genome	1
	2	26795	37	Actinobacillus suis H91-0380 complete genome	1
	3	26643	36	Middle East respiratory syndrome coronavirus Isolate Qatar4 complete genome	12
1501	1	828116	3967	Middle East respiratory syndrome coronavirus complete genome	1
	2	26937	33	Actinobacillus suis H91-0380 complete genome	17
	3	26761	34	PREDICTED: Equus caballus uncharacterized LOC102148405 (LOC102148405)	1

Samples 819 and 820, from the gastrointestinal tract of the bat species *Microchiroptera*, contain a coronavirus. The pipeline analysis on the non-host reads of 819 produced 12 contigs, but only one contig has a match in the NCBI GenBank database. A query of this contig produced a match for Zaria bat coronavirus strain ZBCoV [13]. The reads that failed to map to ZBCoV still produced contigs in the next iteration. No database hits were found for these contigs, so the iterative pipeline analysis was terminated. Sample 820 had a hit against bat host reads in the first iteration, but successfully assembled a contig matching to the Zaria bat coronavirus during the second iteration (Table 3). The other 36 contigs assembled did not have any hits in the database. The iterative pathogen identification analysis is completed after two cycles for this sample due to the lack of contigs mapping to any known pathogens.

Samples 1500 and 1501 were nasal swabs from dromedaries in Saudi Arabia [18] where RNA was isolated and cDNA made from the RNA directly present within the swab. The samples were then sequenced using the Ion Torrent PGM platform. These data generated a large percentage of non-host reads (>90 %), most likely due to high viral loads within the nasal cavities. The iterative analysis efficiently processed the Ion Torrent data, and MERS-CoV was the nearest neighbor identified during the first iteration in both samples (Table 3). One contig was generated from the mapped reads to the identified nearest neighbor (MERS-CoV) reference, and this led to the subtraction of a majority of the reads hitting against MERS-CoV for subsequent novel contig generation.

#### Pathogens detected through taxonomy analysis

The iterative analysis pipeline was unable to properly process sample 28 due to the low number of reads. Only 2 % of the original reads are identified as non-host reads, and those non-host reads are assembled into 2 contigs. A BLAST analysis of the contigs did not identify any near neighbors. Therefore the iterative analysis did not report a pathogen in this sample. In the follow-up analysis adding the taxonomy preprocessing step, the taxonomy analysis revealed that only 6 reads are assigned to the expected GBV-D virus [14, 34]. Unlike the negative control sample, the pathogen of interest is clearly identified through this analysis. The viral and bacterial reads are described in the short report produced by this analysis, and the user would be provided a clear indication of the pathogen in the sample for follow-on analysis.

#### Samples that demonstrate gaps

In the case of sample 1164, multiple contigs were constructed, and the software identified several near neighbors during the iterative analysis. However, in each iteration, the near neighbor identified was mammalian, with most hits mapping to other bat species. No parvovirus hits were found for the contigs, despite the sample being confirmed as parvovirus positive. Using CUPP output as input to the taxonomy analysis, the pathogen was still not identified through the iterative analysis. Unlike all other samples evaluated here, none of the reads derived from the pathogen could be identified by blastn. The pathogen present in this sample was not similar enough to match to anything in the nucleic acid reference database using the search parameters built into the pipeline.

### Analysis of unknown samples not detected by iterative analysis using USAMRIID-WRAIR pipeline

Pathosphere was designed to host multiple analytical pipelines at once, especially as newer technologies and approaches emerge. This capability is demonstrated by using an NGS analytical pipeline designed by the Comparative Genomics Sciences group at the United States Army Medical Research Institute of Infectious Disease (USAMRIID) and the Viral Diseases Branch at Walter Reed Army Institute of Research (WRAIR). The USAMRIID-WRAIR pipeline is available on Pathosphere via the Pathogen Detection Tool, and was used to reanalyze the datasets corresponding to the samples containing GBV-D virus (Sample 28) and parvovirus (sample 1164) (Table 1). The architecture of the USAMRIID-WRAIR pipeline differs from the iterative analysis pipeline tested above (see methods) and reports on the individual read and contig identification and BLAST mapping comparison. Utilizing the USAMRIID-WRAIR pipeline to reanalyze samples 28 and 1164, Pathosphere yielded identification of viral reads matching to the pathogen in both samples (Table 4). Sample 28 yielded 3 viral reads out of 692 BLAST identified non-host reads, all matching to GBV-D virus. Sample 1164 had 24 viral reads mapping to uncultured marine virus, gray fox amdovirus (parvovirus), aeromonas phage, and multiple herpesviruses out of 4621 total BLAST identified reads. Each of the correct pathogen reads are represented in Table 4. The read mapping to parvovirus was identified, but 23 other reads mapped to viruses that could potentially be in the sample. The USAMRIID-WRAIR pipeline offered a different analytical approach, identifying 3 GBV-D reads in sample 28 while identifying a single read mapping to a divergent parvovirus.

**Table 4:** USAMRIID-WRAIR pipeline reanalysis for pathogen reads from Sample 28, 1164. Pathosphere’s ability to host multiple pipelines was tested using a pipeline designed by USAMRIID and WRAIR to analyze datasets from samples 28 and 1164. The reanalysis resulted in viral hits against the correct agent (sample 28) and against multiple viruses (sample 1164) with one correctly identified as a nearest neighbor

Reanalyzed Sample	Read length	NN hit
Sample 28	152	GB virus D strain 93 polyprotein precursor, gene, partial cds
692 total hits		
	231	GB virus D strain 93 polyprotein precursor, gene, partial cds
3 viral hits		
	246	GB virus D strain 93 polyprotein precursor, gene, partial cds
Sample 1164	171	Gray fox amdovirus NS1, NS2, NS3, VP1, and VP2 genes, complete_cds
4621 total hits		
24 viral hits		

## Discussion

To compare the analysis capabilities of pipelines hosted by Pathosphere, spiked human samples were first sequenced using 454, Ion Torrent, and Illumina platforms. Each dataset was then analyzed by the ECBC-developed pipelines hosted on Pathosphere (Table 1). The pipelines had no issues identifying the more abundant pathogens in each dataset through both taxonomic assignment and iterative assembly. The taxonomic assignment is read-based classification, while the iterative assembly is a process that generates contigs and then maps reads against those contigs within the dataset. Read-based taxonomic classification is useful, especially when looking at lower levels of pathogens. This was illustrated in the 454 and Illumina datasets, where only the taxonomic classification identified the *Burkholderia* species in low abundance. Iterative assembly is a mechanism that allows for greater confidence in pathogen ID within a dataset of interest, as contigs are generated that cover a greater percentage of the genome than single reads. Overall, the ECBC pipelines performed well when analyzing low to intermediate levels of spiked pathogens after sequencing on multiple platforms.

Pathosphere analysis of NGS datasets containing pathogens within complex sample backgrounds resulted in positive identification for each sample (Table 3 and 4). However, the two samples with pathogens only detected in read-level taxonomy analysis represent critical sample processing and analysis gaps for how pathogens are detected in NGS samples. Sample 28, which contained GBV-D [14], was identified using the taxonomic analysis but not the iterative analysis due to a low number of reads. Utilizing another analytical pipeline hosted on Pathosphere, built by USAMRIID-WRAIR, allowed for the detection in a sample with a single read mapping to a parvovirus (Table 4). The low levels of pathogens present in many sample types can prevent contig assembly and mapping, but the taxonomy preprocessing tool and the USAMRIID-WRAIR pipeline can lead to successful pathogen identification. In cases of low pathogen load, the detection of any reads mapping to the actual virus within the sample can be extremely useful for pathogen detection. However, better sampling processing methods and tools to evaluate pathogens at lower read levels must be developed to automate the detection of pathogens at low-levels of infectivity.

The absence of parvovirus-matching sequence in the reads from sample 1164 using the iterative assembly and taxonomy preprocessing highlights one of the major challenges facing sequence-based identification of pathogens; how to detect a pathogen with little or no homology to something already deposited into an available database? The use of other methods to identify homology when the NCBI nt database does not have adequate representation available will be the second major area of improvement for the analytical pipelines used in this study. Further investigation of sample 1164 revealed that, by using the discontiguous megablast searching algorithm against the NCBI nt database, or using blastx against the NCBI nr database, many reads show matches to parvovirus records. The issue revealed by samples like 1164 is a major focus of the field going forward, and is something that can be addressed through Pathosphere as novel tools for pathogen discovery are developed.

Most pathogens identified to date have had some sequence homology to previously identified pathogens, so it is very possible that etiological agents are being missed due to the lack of homology to existing known sequences [35]. Further, pathogen detection accuracy is important as environmental or sample contamination can lead to false pathogen discovery [36]. With increasing biosurveillance efforts in human and non-human populations, there will be large amounts of data generated that potentially contain novel pathogens with little or no homology to existing viruses [37, 38]. Less stringent nucleotide alignment approaches and BLASTx (translated nucleotide sequence to amino acid sequence) have the potential to detect some of these less homologous pathogens. However, many of these algorithms, such as BLASTx, are too computationally intensive to implement without heuristics or substantial dedicated computational resources. As more efficient algorithms are developed, such as the newly described DIAMOND [39], they can be integrated into the existing analytical pipelines as well as pipelines developed elsewhere and hosted on Pathosphere. The current pipelines bin total contiguous sequences for the user to analyze further. This provides an area for future development, as identifying unknown contigs remains a critical area for pathogen identification and discovery. Pathosphere provides the necessary architecture to host the types of software programs that in the future will be needed to analyze data sets for unknown pathogens that contain little or no homology to pathogens described previously. Providing the source code for the preprocessing tools (http://sourceforge.net/projects/pathosphere) as well as the raw data sets utilized here (via Pathosphere) provides a standard starting point for the further evaluation of these pipelines as well as the integration of new tools into Pathosphere.

Sequencer platform of choice also plays a role in using NGS as a tool for pathogen detection. Platforms like Ion Torrent will give longer read lengths, while Illumina technology will give shorter read lengths (making contig assembly more complex) but can provide greater depth and coverage of all the genomic material present in a given sample [40]. Technology from PacBio generates very long read length, making contig assembly less important. Novel sequencing technology, such as portable nanopore sequencing, would benefit from centralized analytical tools that can be accessed and utilized remotely [41, 42]. The ability of the pipelines tested here to detect pathogens in both 454, Ion Torrent, and Illumina sequenced samples demonstrates the utility of Pathosphere to host pipelines meant for analysis of data from different platforms. As tools become available to better match reads to databases and to analyze data from varying sequencing platforms, the plug-in support of Pathosphere will allow for the integration of these tools into the analytical pipelines.

The real-time detection of pathogens is an important step for more complete biosurveillance efforts worldwide and is critical when responding to an outbreak of unknown origin. Collaborative crowd-sourcing has emerged as a tool to quickly identify pathogens during outbreaks, like during the enterohemorrhagic *E. coli* outbreak in Europe during 2011 [43, 44]. Despite this collaboration, determining strain level identification of certain agents from NGS datasets remains a major gap, although the field is creating tools (that could be hosted on Pathosphere) utilizing multiple read-level loci for strain identification (One Codex, Pathoscope [7, 45, 46]). This becomes a greater challenge when the mechanisms of virulence are unknown, as the strain differences between the well-characterized O104:H4 EHEC E. coli and other less pathogenic strains are still being explored [47, 48]. Similar efforts in the future will benefit from the centralized and adaptable analysis hub provided by Pathosphere.

Pathosphere differs from already available services like Galaxy [49] because its primary focus is the detection of pathogens in complex samples. Tools have been developed for pathogen detection in NGS datasets, such as SURPI [3], but these tools are not hosted. Further, Pathosphere offers accessibility to bioinformatics software for users not familiar with these tools, which is a major gap in using NGS for public health applications and for guiding clinical diagnostic procedures [50, 51]. The pipelines hosted are designed only for that purpose, and the variable outputs from these pipelines can range from simple taxonomy and contig ID reports to more bioinformatically-intensive single read alignment files. This creates a pathogen-centric approach to sequencing data analysis that serves to focus both experienced and inexperienced users. Pathosphere provides these services through a user-friendly, web-based portal that pulls data uploaded by researchers and performs the desired analyses using hardware supported remotely. The analysis reports are then communicated back to the user via Pathosphere email alerts; and the pathogens contained within the sequence data can be identified. This setup can be advantageous in many situations, especially when the costly resources needed to run these analyses locally are unavailable or when the environment the data is collected in might not be optimal for software hosting [52].

In addition to the pipeline analysis presented here, the hosting architecture of Pathosphere has already had extensive peer use. Pathosphere has a user base of over 150 individuals from organizations such as the CDC, the Department of Defense, MIT, Columbia University, and organizations based internationally. Software available on Pathosphere is hosted in collaboration with MITLL, the University of Houston, and OptiMetrics. Pathosphere has been used by the community to facilitate international collaboration, and was instrumental in the genomic analysis of novel enterovirus isolates in South America [53]. The current Ebola outbreak has highlighted the need for available tools for infectious disease personnel on the ground in western Africa [54, 55]. As the epidemic becomes more controlled and more personnel are established, genomic surveillance and molecular epidemiology will become key to understanding the dynamics of the current epidemic as well as to provide information for the prevention of the next ebolavirus epidemic [56–59]. The remote capabilities of Pathosphere could help fill these needs and remove the need for IT personnel, bioinformatics specialists, and computing hardware at the epicenter of an outbreak.

## Conclusions

Pathosphere supports the evaluation of novel detection algorithms and other analytical tools by allowing users to run these potentially process-heavy applications using the hardware that supports the web interface. The users of Pathosphere can communicate directly with the technical development team through forums and discussion boards on the web interface. This ongoing collaboration between Pathosphere developers and users ensures that the most current and accurate ways to detect pathogens in traditional and NGS data are utilized in the analytical pipelines. The user-friendly features (including communication methods) built into Pathosphere, its utility for detecting pathogens in complex samples, and its plug-in development architecture allow for it to evolve with novel technology and provide a comprehensive web interface for the detection of known pathogens and emerging infectious diseases worldwide.

## Additional file

Additional file 1: (zip 23 kb)

## References

[1] Leopold SR, Goering RV, Witten A, Harmsen D, Mellmann A (2014). Bacterial whole genome sequencing revisited: portable, scalable and standardized analysis for typing and detection of virulence and antibiotic resistance genes. J Clin Microbiol.

[2] Manary MJ, Singhakul SS, Flannery EL, Bopp SE, Corey VC, Bright AT (2014). Identification of pathogen genomic variants through an integrated pipeline. BMC Bioinformatics.

[3] Naccache SN, Federman S, Veeraraghavan N, Zaharia M, Lee D, Samayoa E (2014). A cloud-compatible bioinformatics pipeline for ultrarapid pathogen identification from next-generation sequencing of clinical samples. Genome Res.

[4] Lipkin WI (2013). The changing face of pathogen discovery and surveillance. Nat Rev Microbiol.

[5] Deng X, Naccache SN, Ng T, Federman S, Li L, Chiu CY (2015). An ensemble strategy that significantly improves de novo assembly of microbial genomes from metagenomic next-generation sequencing data. Nucleic Acids Res.

[6] D’Auria G, Schneider MV, Moya A (2014). Live genomics for pathogen monitoring in public health. Pathog.

[7] Byrd AL, Perez-Rogers JF, Manimaran S, Castro-Nallar E, Toma I, McCaffrey T (2014). Clinical PathoScope: rapid alignment and filtration for accurate pathogen identification in clinical samples using unassembled sequencing data. BMC Bioinformatics.

[8] Freitas TAK, Li P-E, Scholz MB, Chain PSG (2015). Accurate read-based metagenome characterization using a hierarchical suite of unique signatures. Nucleic Acids Res.

[9] Fusaro VA, Patil P, Gafni E, Wall DP, Tonellato PJ (2011). Biomedical cloud computing with Amazon Web Services. PLoS Comput Biol.

[10] Briese T, Paweska JT, McMullan LK, Hutchison SK, Street C, Palacios G (2009). Genetic detection and characterization of Lujo virus, a new hemorrhagic fever-associated arenavirus from southern Africa. PLoS Pathog.

[11] Bohlander SK, Espinosa R, Le Beau MM, Rowley JD, Díaz MO (1992). A method for the rapid sequence-independent amplification of microdissected chromosomal material. Genomics.

[12] Palacios G, Quan P, Jabado OJ, Conlan S, Hirschberg DL, Liu Y (2007). Panmicrobial oligonucleotide array for diagnosis of infectious diseases. Emerg Infect Dis.

[13] Quan P-LL, Firth C, Street C, Henriquez JA, Petrosov A, Tashmukhamedova A (2010). Identification of a severe acute respiratory syndrome coronavirus-like virus in a leaf-nosed bat in Nigeria. MBio.

[14] Epstein JH, Quan P-L, Briese T, Street C, Jabado O, Conlan S (2010). Identification of GBV-D, a novel GB-like Flavivirus from Old world frugivorous bats (pteropus giganteus) in bangladesh. PLoS Pathog.

[15] Kapoor A, Simmonds P, Lipkin WI (2010). Discovery and characterization of mammalian endogenous parvoviruses. J Virol.

[16] Canuti M, Eis-Huebinger AM, Deijs M, de Vries M, Drexler JF, Oppong SK (2011). Two novel parvoviruses in frugivorous New and Old world bats. PLoS One.

[17] Alagaili AN, Briese T, Mishra N, Kapoor V, Sameroff SC, de Wit E, Munster VJ, Hensley LE, Zalmout IS, Kapoor A, Epstein JH, Karesh WB, Daszak P, Mohammed OB, Lipkin WI: Middle East Respiratory Syndrome Coronavirus Infection in Dromedary Camels in Saudi Arabia. *MBio* 2014, 5:e00884–14–e00884–14. 10.1128/mBio.00884-14 PMC3940034 24570370

[18] Briese T, Mishra N, Jain K, Zalmout IS, Jabado OJ, Karesh WB (2014). Middle East respiratory syndrome coronavirus quasispecies that include homologues of human isolates revealed through whole-genome analysis and virus cultured from dromedary camels in Saudi Arabia. MBio.

[19] Cotten M, Lam TT, Watson SJ, Palser AL, Petrova V, Grant P, Pybus OG, Rambaut A, Guan Y, Pillay D, Kellam P, Nastouli E: Full-genome deep sequencing and phylogenetic analysis of novel human betacoronavirus. Emerg Infect Dis 2013, 19:10.3201/eid1905.130057. 10.3201/eid1905.130057 PMC3647518 23693015

[20] Koskiniemi S, Gibbons HS, Sandegren L, Anwar N, Ouellette G, Broomall S (2013). Pathoadaptive mutations in Salmonella enterica isolated after serial passage in mice. PLoS One.

[21] Kapoor A, Simmonds P, Cullen JM, Scheel TKH, Medina JL, Giannitti F (2013). Identification of a pegivirus (GB virus-like virus) that infects horses. J Virol.

[22] Langmead B, Trapnell C, Pop M, Salzberg SL (2009). Ultrafast and memory-efficient alignment of short DNA sequences to the human genome. Genome Biol.

[23] Miller JR, Koren S, Sutton G (2010). Assembly algorithms for next-generation sequencing data. Genomics.

[24] Zerbino DR, Birney E (2008). Velvet: algorithms for de novo short read assembly using de Bruijn graphs. Genome Res.

[25] Langmead B, Salzberg SL (2012). Fast gapped-read alignment with Bowtie 2. Nat Methods.

[26] Martin M (2011). Cutadapt removes adapter sequences from high-throughput sequencing reads. EMBnet J.

[27] Schmieder R, Edwards R (2011). Quality control and preprocessing of metagenomic datasets. Bioinformatics.

[28] Boisvert S, Raymond F, Godzaridis E, Laviolette F, Corbeil J (2012). Ray Meta: scalable de novo metagenome assembly and profiling. Genome Biol.

[29] Huang X, Madan A (1999). CAP3: A DNA sequence assembly program. Genome Res.

[30] Fournier P-E, Drancourt M, Colson P, Rolain J-M, La Scola B, Raoult D (2013). Modern clinical microbiology: new challenges and solutions. Nat Rev Microbiol.

[31] Padmanabhan R, Mishra AK, Raoult D, Fournier P-E (2013). Genomics and metagenomics in medical microbiology. J Microbiol Methods.

[32] Biesbroek G, Sanders EAM, Roeselers G, Wang X, Caspers MPM, Trzciński K (2012). Deep sequencing analyses of low density microbial communities: working at the boundary of accurate microbiota detection. PLoS One.

[33] Paweska JT, Sewlall NH, Ksiazek TG, Blumberg LH, Hale MJ, Lipkin WI (2009). Nosocomial outbreak of novel arenavirus infection, southern Africa. Emerg Infect Dis.

[34] Stapleton JT, Foung S, Muerhoff AS, Bukh J, Simmonds P (2011). The GB viruses: a review and proposed classification of GBV-A, GBV-C (HGV), and GBV-D in genus Pegivirus within the family Flaviviridae. J Gen Virol.

[35] Chiu CY (2013). Viral pathogen discovery. Curr Opin Microbiol.

[36] Naccache SN, Greninger AL, Lee D, Coffey LL, Phan T, Rein-Weston A (2013). The perils of pathogen discovery: origin of a novel parvovirus-like hybrid genome traced to nucleic Acid extraction spin columns. J Virol.

[37] Lipkin WI, Firth C (2013). Viral surveillance and discovery. Curr Opin Virol.

[38] Levinson J, Bogich TL, Olival KJ, Epstein JH, Johnson CK, Karesh W (2013). Targeting surveillance for zoonotic virus discovery. Emerg Infect Dis.

[39] Buchfink B, Xie C, Huson DH: Fast and sensitive protein alignment using DIAMOND. Nat Methods 2014. 12(1):59-60. doi:10.1038/nmeth.3176. 10.1038/nmeth.3176 25402007

[40] Frey KG, Herrera-Galeano JE, Redden CL, Luu TV, Servetas SL, Mateczun AJ (2014). Comparison of three next-generation sequencing platforms for metagenomic sequencing and identification of pathogens in blood. BMC Genomics.

[41] Kilianski A, Haas JL, Corriveau EJ, Liem AT, Willis KL, Kadavy DR (2015). Bacterial and viral identification and differentiation by amplicon sequencing on the MinION nanopore sequencer. Gigascience.

[42] Quick J, Ashton P, Calus S, Chatt C, Gossain S, Hawker J (2015). Rapid draft sequencing and real-time nanopore sequencing in a hospital outbreak of Salmonella. Genome Biol.

[43] Mellmann A, Harmsen D, Cummings CA, Zentz EB, Leopold SR, Rico A (2011). Prospective genomic characterization of the German enterohemorrhagic Escherichia coli O104:H4 outbreak by rapid next generation sequencing technology. PLoS One.

[44] Rohde H, Qin J, Cui Y, Li D, Loman NJ, Hentschke M (2011). Open-source genomic analysis of Shiga-toxin-producing E. coli O104:H4. N Engl J Med.

[45] Francis OE, Bendall M, Manimaran S, Hong C, Clement NL, Castro-Nallar E (2013). Pathoscope: species identification and strain attribution with unassembled sequencing data. Genome Res.

[46] Hong C, Manimaran S, Shen Y, Perez-Rogers JF, Byrd AL, Castro-Nallar E (2014). PathoScope 2.0: a complete computational framework for strain identification in environmental or clinical sequencing samples. Microbiome.

[47] Boisen N, Hansen A-M, Melton-Celsa AR, Zangari T, Mortensen NP, Kaper JB (2014). The presence of the pAA plasmid in the German O104:H4 Shiga toxin type 2a (Stx2a)-producing enteroaggregative Escherichia coli strain promotes the translocation of Stx2a across an epithelial cell monolayer. J Infect Dis.

[48] Kunsmann L, Rüter C, Bauwens A, Greune L, Glüder M, Kemper B (2015). Virulence from vesicles: Novel mechanisms of host cell injury by Escherichia coli O104:H4 outbreak strain. Sci Rep.

[49] Goecks J, Nekrutenko A, Taylor J (2010). Galaxy: a comprehensive approach for supporting accessible, reproducible, and transparent computational research in the life sciences. Genome Biol.

[50] Loeffelholz M, Fofanov Y: The main challenges that remain in applying high-throughput sequencing to clinical diagnostics. Expert Rev Mol Diagn 2015;15(11):1405-8. doi:10.1586/14737159.2015.1088385. 10.1586/14737159.2015.1088385 26394651

[51] Grad YH, Lipsitch M (2014). Epidemiologic data and pathogen genome sequences: a powerful synergy for public health. Genome Biol.

[52] Lim YW, Cuevas DA, Silva GGZ, Aguinaldo K, Dinsdale EA, Haas AF (2014). Sequencing at sea: challenges and experiences in Ion Torrent PGM sequencing during the 2013 Southern Line Islands Research Expedition. PeerJ.

[53] Tokarz R, Hirschberg DL, Sameroff S, Haq S, Luna G, Bennett AJ (2013). Genomic analysis of two novel human enterovirus C genotypes found in respiratory samples from Peru. J Gen Virol.

[54] Jacob ST, Crozier I, Schieffelin JS, Colebunders R (2014). Priorities for Ebola virus disease response in west Africa. Lancet.

[55] Vogel G (2014). Infectious Diseases. Delays hinder Ebola genomics. Science.

[56] Gire SK, Goba A, Andersen KG, Sealfon RSG, Park DJ, Kanneh L (2014). Genomic surveillance elucidates Ebola virus origin and transmission during the 2014 outbreak. Science.

[57] Kugelman JR, Wiley MR, Mate S, Ladner JT, Beitzel B, Fakoli L (2015). Monitoring of Ebola Virus Makona Evolution through Establishment of Advanced Genomic Capability in Liberia. Emerg Infect Dis.

[58] Park DJ, Dudas G, Wohl S, Goba A, Whitmer SLM, Andersen KG (2015). Ebola Virus Epidemiology, Transmission, and Evolution during Seven Months in Sierra Leone. Cell.

[59] Hoenen T, Safronetz D, Groseth A, Wollenberg KR, Koita OA, Diarra B (2015). Virology, Mutation rate and genotype variation of Ebola virus from Mali case sequences. Science.

